# In Vivo Gastroprotective Upshots of the Novel Schiff Base CdCl2 (C14H21N3O2) Compound by Bax/HSP-70 Signaling and Inflammatory Cytokines

**DOI:** 10.7759/cureus.75963

**Published:** 2024-12-18

**Authors:** Suhayla H Shareef

**Affiliations:** 1 Department of Biology, College of Education, Salahaddin University-Erbil, Erbil, IRQ

**Keywords:** bax, gastric ulcer, histopathology, hsp-70, il-10, il-6, immunohistochemistry, novel schiff base cdcl2 (c14h21n3o2) compound, tnf-α

## Abstract

Background: Synthesis of the original Schiff base CdCl_2 _(C_14_H_21_N_3_O_2_) compound (Schiff base complex) displays an extensive range of bioactivities and was predictably utilized to treat several syndromes.

Purpose: The goal of the existing experiment is to evaluate the gastroprotective effects of a novel Schiff base CdCl₂ (C14H21N3O2) compound in alcohol-induced gastric ulcers in rats by examining its antioxidant activity, anti-inflammatory effects, and modulation of key molecular markers, including heat shock protein-70 (HSP-70) and Bcl-2-associated X protein (Bax) proteins.

Methods: Five groups of rats were utilized in the current study. Control and model groups were orally administered 10% Tween 20. The treated groups were orally administered 20 mg/kg omeprazole or Schiff base compound (25 or 50 mg/kg). One hour later, only the control group received oral 10% Tween 20, and the treated groups received oral (5 ml/cage) absolute alcohol. During the second hour, all rats were sacrificed.

Results: All treated rats presented considerable improvement in alcohol-induced gastric injury recognized by decreasing ulcer index and raising % of ulcer inhibition. Increased mucus and gastric pH content and decreased ulcerated portion, reduced or non-appearance of edema, and leucocytes penetrated the subcutaneous layer. In stomach epithelium homogenate, the Schiff base compound obtainable significant upsurge superoxide dismutase (SOD), catalase (CAT) activities, considerable declining malondialdehyde (MDA) quantity. Moreover, the Schiff base compound raised the intensity of periodic acid-Schiff (PAS) stains gastric epithelium. Furthermore, the Schiff base compound formed up-regulated HSP-70 and down-regulated Bax proteins gastric epithelium Schiff base compound, reduced the level of tumor necrosis factor-alpha (TNF-α), interleukin-6 (IL-6) and improved the quantity of IL-10. Administering a high dose of 500 mg/kg Schiff base compound revealed the nontoxic nature of the compound in rats.

Conclusion: Schiff base compound exhibited gastroprotective effects attributed to its antioxidant nature, its capability to enhance mucus excretion, SOD and CAT, reduce MDA amount, up-regulate HSP-70 protein, down-regulate Bax protein, and inflammatory cytokines.

## Introduction

Gastric ulcer is the highest common gastrointestinal pathology and disruption of the mucosal fence of gastric coating affecting up to 10%-15% of people in the general population. Gastric ulcer, manifest by the erosion of the gastrointestinal mucosa, represents a significant clinical challenge in gastroenterology and accompanied by stomach ache, sickness, and hemorrhage puncture [1,2].

Peptic ulceration disease is the chief gastric system syndrome globally. Numerous studies have shown disorder, a disproportion between mucosal aggressive issues, namely, *Helicobacter pylori* infection, ingesting of non-steroidal anti-inflammatory drugs, stress, and undernourishment, causing disruption of self-protective stomach epithelial barrier, leading to gastrointestinal ulceration, and self-protective parts gastric epithelial barrier comprise mucus secretion, increased prostaglandin amount, and endogenous antioxidant enzyme action [3,4].

Absolute alcohol is a damaging mediator that encourages oxidative tension gastric epithelial damage terminated production of extremely cytotoxic free radicals [5]. Reactive oxygen species (ROS) produced by ethanol gavage amongst epithelial aggressive topics responsible for gastric ulceration by producing oxidative injury of stomach epithelial layers [6]. ROS and oxidants cause injury to stomach epithelial layers. Absolute alcohol upsurges superoxide anion, hydroxyl radical manufacture, and lipid peroxidation stomach epithelium [7]. Furthermore, ethanol disturbs gastric mucus action, variations cell penetrability, and reduces stomach mucus excretion and epithelial cells more susceptible to free radicals [1,2]. Numerous studies revealed oral gavage of ethanol produces morphological variations in the stomach epithelia, for example, decreased stomach hypersecretion, and reduced mucus emission hemorrhagic injuries to the stomach epithelia of treated rats [1,2,4].

A Schiff base is a compound made by condensing a carbonyl-containing aldehyde or ketone with a nitrogen-based counterpart, resulting in the replacement of the carbonyl group with an imine group called azomethine. Schiff bases and their complexes have attracted significant attention in the field of coordination chemistry and have become well known for their extensive biological potential [8]. In general, Schiff bases are stable solids and can be stored without many precautions. A variety of biological actions, including anti-HIV, antibacterial, antifungal , herbicidal, anti-tubercular, anti-diabetic, anti-cancer, anti-proliferative, and anti-inflammatory activities have been elicited from Schiff metal complexes [8]. Numerous studies on Schiff bases with metal complexes of manganese, nickel, zinc, copper, and cobalt have been reported. CdCl2 (C14H21N3O2) complex is able to induce the apoptosis of colon cancer cells [9]. The research examined gastroprotective effects of the CdCl2 (C14H21N3O2) compound on absolute alcohol-induced stomach injury in rats. Graphical abstract is given in Appendix.

## Materials and methods

Ethical declaration

The research was conducted following the Animal Research Reporting of In Vivo Experiments (ARRIVE) guidelines and in conformity with guidelines confirmed by Iraqi animal rights and National scientific recommendations for laboratory animal experiments [10]. The present animal procedure was approved by the Research Ethics Committee of Cihan University, Erbil Kurdistan region/Iraq with the approval ID number: ERB 89/09/9/2024/MAA.

Constituents and substances

All reagents and compounds were purchased from Sigma-Aldrich, St. Louis, MO, USA, excluding omeprazole purchased from Gastuf 20, Geneda Pharma, India. Omeprazole dissolved by 10% Tween 20 was gavage orally to rats in dosage 20 mg/kg.

Acute toxicity study

The acute toxicity study determined harmless dosage (250 and 500 mg/kg) of Schiff base compound. Thirty-six male and female Sprague Dawley (SD) rats (18 males and 18 females) age (eight weeks), weight (220-240 g) were separated into three identical groups, control, low dosage (250 mg/kg), and high dosage (500 mg/kg) Schiff base compound. Rats were fasted (food not water) for one day. Furthermore, the diet reserved an additional three to four hrs. after treating the compound. For the acute toxicity experiment dosage was chosen consistent with the Institute Financial Co-operation Progress Advice on harmfulness testing [11]. Rats observed 30 min and two, four, eight, 24, and 48 hours after gavage Schiff base compound for symbols toxicity, behavioral abnormality, and rates of mortality. Rats were checked every day for characteristic indications of harmfulness for 15 days. On day 15, rats were euthanized via general anesthesia using ketamine (30 mg/kg, 100 mg/mL) and xylazine (3 mg/kg, 100 mg/mL). Blood collected from intra-cardiac puncture for biochemical parameters. Histology of kidney and liver stain determined by standard method [12].

Animal experimental of gastric ulcer

Male rats were randomly clustered into five groups (each group six) of rats. Rat weighted 220-240 g placed separately in distinct cage wide-net cord bases to avoid coprophagia throughout the experiment. Rats fed usual pellet diet and tap water [13].

Stomach ulcer induction by absolute alcohol

The rats fast (food but not water) 24 hours to empty the stomach prior experiment [1]. Stomach ulceration induced by absolute alcohol (5 ml/kg). G1 (normal control group) and G2 (ulcerated control group) were orally gavage with 10% Tween 20. G3 obtained oral dosages of 20 mg/kg omeprazole 10% Tween 20. G4 and G5 (experimental groups) were gavage with the Schiff base compound at dosages of 25 and 50 mg/kg. One hour afterward pre-treatment G1 rats were gavage with 10% Tween 20, while two to five groups were administered orally absolute alcohol to produce stomach ulceration [14]. Rats were sacrificed an hour subsequently over the dosage of xylazine; ketamine anesthesia stomachs were removed immediately.

Gross evaluation of stomach lesions

Stomach opened lengthways greater curving, and stomach mucosa washed gently with ice-cold-buffered saline. Ulcerated stomach epithelium looks like extended bands of red lacerations. Stomach epithelium inspected for injury. Distance and thickness ulcerated area (mm) resolute via a plan meter (10 × 10 mm^2^ = ulcer area) below the optical microscope (1.8x). The ulcer part was calculated via numeration numeral minor quadrangles, 2 mm × 2 mm, covering the distance, and width ulcerated band. Amount areas altogether lacerations every stomach practical estimate ulcerated area (UA) quantity slight squares × 4 × 1.8 = UA (mm^2^). Inhibition % (I.0 %) is calculated by following the formula [1,3]:

(Inhibition %) = [(Ulcer area control - Ulcer area treated)¸ Ulcer area control] × 100%.

Assessment of stomach mucus content

Gastric mucus construction amount exposed to absolute alcohol produced stomach ulcers. Stomach epithelium gained via gentle rubbing epithelia clean cut-glass gathered mucus balanced utilizing an accurate electrical equilibrium [2].

Measurement of stomach pH 

Specimens of stomach fluid fillings examine hydrogen ion concentration via pH metric titration 0.1N NaOH using an arithmetical pH meter sourness slow via mEq/L [2].

Preparation of gastric tissue homogenate

Minor slice gastric wall from each rat were washed prudently with phosphate-buffered saline (PBS). Utilizing homogenizer, stomach wall slice normalized PBS, including mammalian protease inhibitor combination. Stomach homogenates tissue separator 1000x g 15 min 4°C. Pure supernatant fluids quantity superoxide dismutase (SOD), catalase (CAT), and malondialdehyde (MDA). Assessment achieved conferring manufacturer’s advice [15].

Assessment antioxidant enzymes activity

SOD and CAT activities of the stomach homogenate were measured using commercial standard kits (Cayman Chemical Co., Ann Arbor, USA). The manufacturer’s instructions were used to measure their amounts in the homogenate supernatant [12,16].

Measures of malondialdehyde (MDA) amount

MDA level in the glandular gastric homogenate was measured using marketable kits (Cayman Chemical Co., Ann Arbor, USA) conferring to the manufacturer’s instructions [1].

Histopathological assessment of stomach abrasions

Small slices (1-2 cm) of each stomach glandular epithelium were fixed immediately in 10% buffered formalin solution at room temperature for 24 h, followed by tissue dehydration with ethanol, clearance with xylene, and infiltration with paraffin using a tissue processing machine. Each tissue biopsy was embedded in paraffin and sliced into sections of 5 µm thickness (Leica Rotation Microtome, Germany). Sections stained with hematoxylin and eosin stain (H&E) stain and also with periodic acid-Schiff (PAS) stain to differentiate the acidic and basic glycoproteins level in the mucus. To evaluate the mucus secretion of the stomach’s glandular epithelium, stomach slices stained with PAS were evaluated using Image J software [1,17].

Immunohistochemically staining

Stomach slices of 3-5 µm thickness stained via immunostaining using ARKTM (Animal Research Kit) were used to detect the immune-histochemical restrict of heat shock protein-70 (HSP 70) (1:100) and Bcl-2-associated X protein (Bax) (1:50) proteins. The proteins were obtained from Santa Cruz Biotechnology (Dallas, Texas) [1]. Immunohistochemistry-stained positive cell investigation was shown via account positive cells applying microscope. Microscopical pictures assessed utilizing Image J software measure intensity stains. Proportion-positive cells were assessed.

Assessment of cytokines (TNF-α, IL-6, and IL-10)

Estimation of TNF-α, IL-6, and IL-10 in gastric tissue was achieved by using an ELISA kit. This was measured by following the manufacturer’s instructions, stated in the Rat TNF-α ELISA Kit (MBS267737) and the Rat IL-6 ELISA Kit (MBS355410). Cytokine strength measurement was showed via “normal sanitized recombinant cytokines” [2,11,12].

Statistical analysis

Statistical analysis of variance among groups evaluated by using the ANOVA test shadowed by Turkey’s post hoc test. Normality test was achieved by the Kolmogorov-Smirnov test. Data were presented as mean ± SEM. A value of p<0.05 was considered statistical significance.

## Results

Evaluation of acute toxicity study

**Figure 1:**
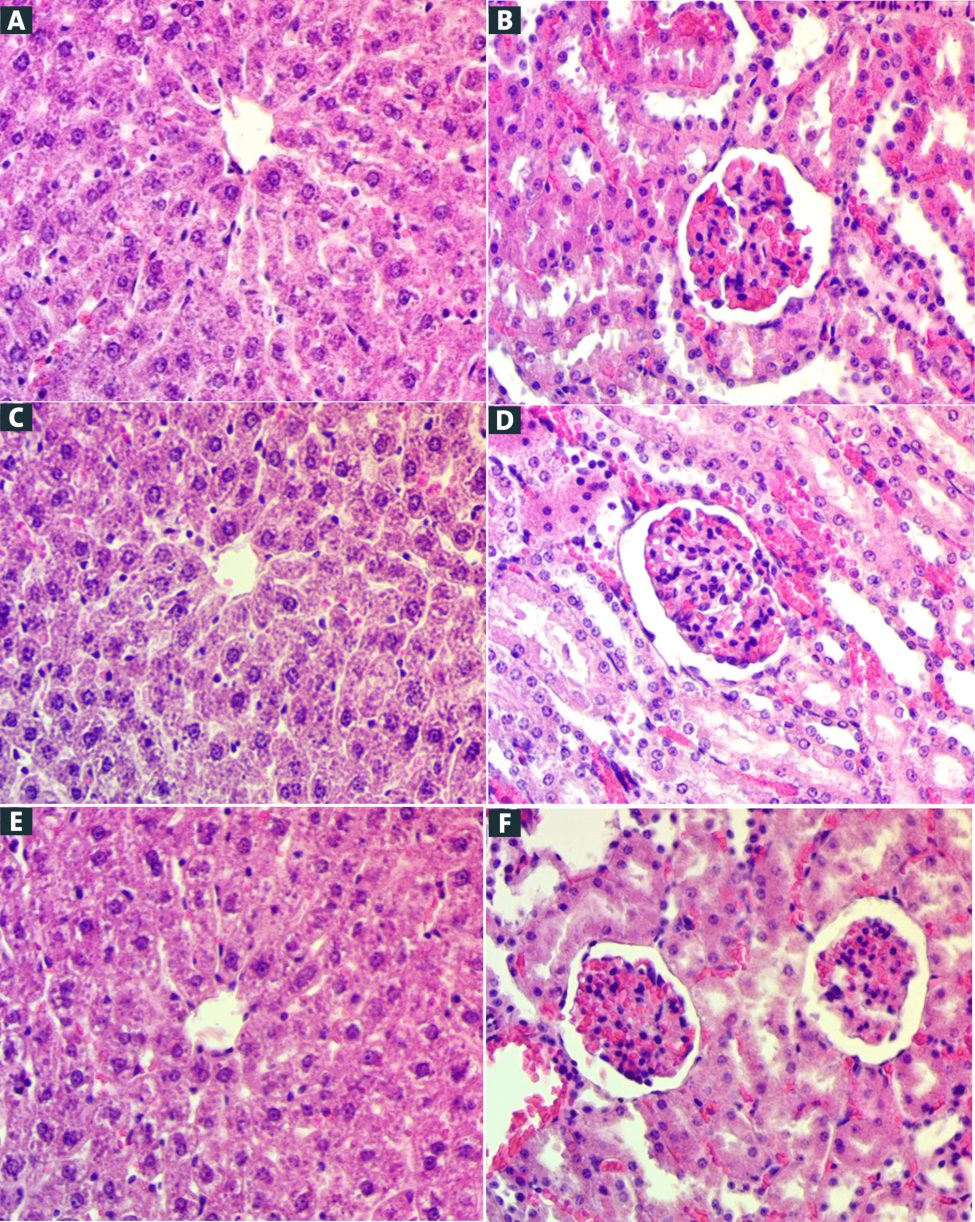
Histology slices of the liver (A, C, and E) and kidney (B, D, and F) for acute toxicity assessment. Rats administered 5 ml/kg 10% Tween 20 (A, B); animals administered Schiff base compound (250 mg/kg) (C, D); and rats administered Schiff base compound (500 mg/kg) (E, F). There are no substantial changes detected in the constructions of the liver and kidney (hematoxylin and eosin (H&E) stains, amplification ×40).

The influence of the Schiff base compound on kidney, liver functions, and lipids profiles in rats did not reveal any sign of toxicity. Rats administered the Schiff base compound showed no evidence of death and harm, as confirmed by renal, liver functions, and lipids profile parameters investigation compared to the control group. Furthermore, a histopathological examination of the liver and kidney and blood biochemical markers showed no considerable changes among the various groups of the male and female rats (Figures 1A-1E and Tables 1-4). Based on the findings of the current study, Schiff base compound was not toxic at either dosage.

**Table 1:** Effects of Schiff base CdCl2 (C14H21N3O2) on kidney biochemical parameters in male rats. Values expressed as mean ± SEM. There are no significant changes between groups. Significant value at p <0.05.

Dose Male	Sodium (mmol/L)	Potassium (mmol/L)	Chloride (mmol/L)	Urea (mmol/L)	Creatinine (μmol/L)
Control (10% Tween 20)	144.8±0.47	5.42±0.09	105.5±0.42	5.19±0.02	31.5±0.76
Schiff base CdCl_2_ (C_14_H_21_N_3_O_2_) (250 mg/kg)	144.5±0.42	5.20±0.15	106.0±0.25	5.35±0.07	30.5±0.76
Schiff base CdCl_2_ (C_14_H_21_N_3_O_2_) (500 mg/kg)	143.7±0.33	5.49±0.15	105.0±0.36	5.40±0.07	32.6±0.66

**Table 2:** Effects of Schiff base CdCl2 (C14H21N3O2) on kidney biochemical parameters in female rats. Values expressed as mean ± SEM. There are no significant changes between groups. Significant value at p <0.05.

Dose Female	Sodium (mmol/L)	Potassium (mmol/L)	Chloride (mmol/L)	Urea (mmol/L)	Creatinine (μmol/L)
Control (10% Tween 20)	148.2±0.47	5.10±0.15	108.9±0.27	5.40±0.13	38.7±0.54
Schiff base CdCl_2_ (C_14_H_21_N_3_O_2_) 250 mg/kg)	149.5±0.67	5.0±0.06	108.4±0.22	5.67±0.08	40.4±0.56
Schiff base CdCl_2_ (C_14_H_21_N_3_O_2_) (500 mg/kg)	148.8±0.70	5.0±0.18	109.1±0.27	5.64±0.09	39.0±0.31

**Table 3:** Effects of Schiff base CdCl2 (C14H21N3O2) on liver biochemical parameters in male rats. Values expressed as mean ± SEM. There are no significant changes between groups. Significant value at p <0.05. ALP: alkaline phosphatase; ALT: alanine aminotransferase; AST: aspartate aminotransferase.

Dose Male	ALP (IU/L)	ALT (IU/L)	AST (IU/L)	Albumin (g/L)	Total Protein (g/L)
Control (10% Tween 20)	108.5±0.76	51.6±0.76	213.0±0.57	13.3±0.33	80.8±0.79
Schiff base CdCl_2_ (C_14_H_21_N_3_O_2_) (250 mg/kg)	109.5±0.42	48.8±0.79	211.3±0.49	13.1±0.30	81.8±0.47
Schiff base CdCl_2_ (C_14_H_21_N_3_O_2_) (500 mg/kg)	107.8±0.70	50.8±0.70	212.8±0.30	12.8±0.30	79.8±0.60

**Table 4:** Effects of Schiff base CdCl2 (C14H21N3O2) on liver biochemical parameters in female rats. Values expressed as mean ± SEM. There are no significant changes between groups. Significant value at p <0.05. ALP: alkaline phosphatase; ALT: alanine aminotransferase; AST: aspartate aminotransferase.

Dose Female	ALP (IU/L)	ALT (IU/L)	AST (IU/L)	Albumin (g/L)	Total Protein (g/L)
Control (10% Tween 20)	108.5±0.54	52.40±0.58	215.2±0.60	13.4±0.54	81.0±0.36
Schiff base CdCl_2_ (C_14_H_21_N_3_O_2_) (250 mg/kg)	118.1±10.55	50.33±0.99	216.2±0.47	14.3±0.56	80.3±0.66
Schiff base CdCl_2_ (C_14_H_21_N_3_O_2_) (500 mg/kg)	106.2±0.65	51.76±0.70	215.0±0.44	12.8±0.47	79.2±0.44

Macroscopic evaluation of stomach lesions

**Figure 2:**
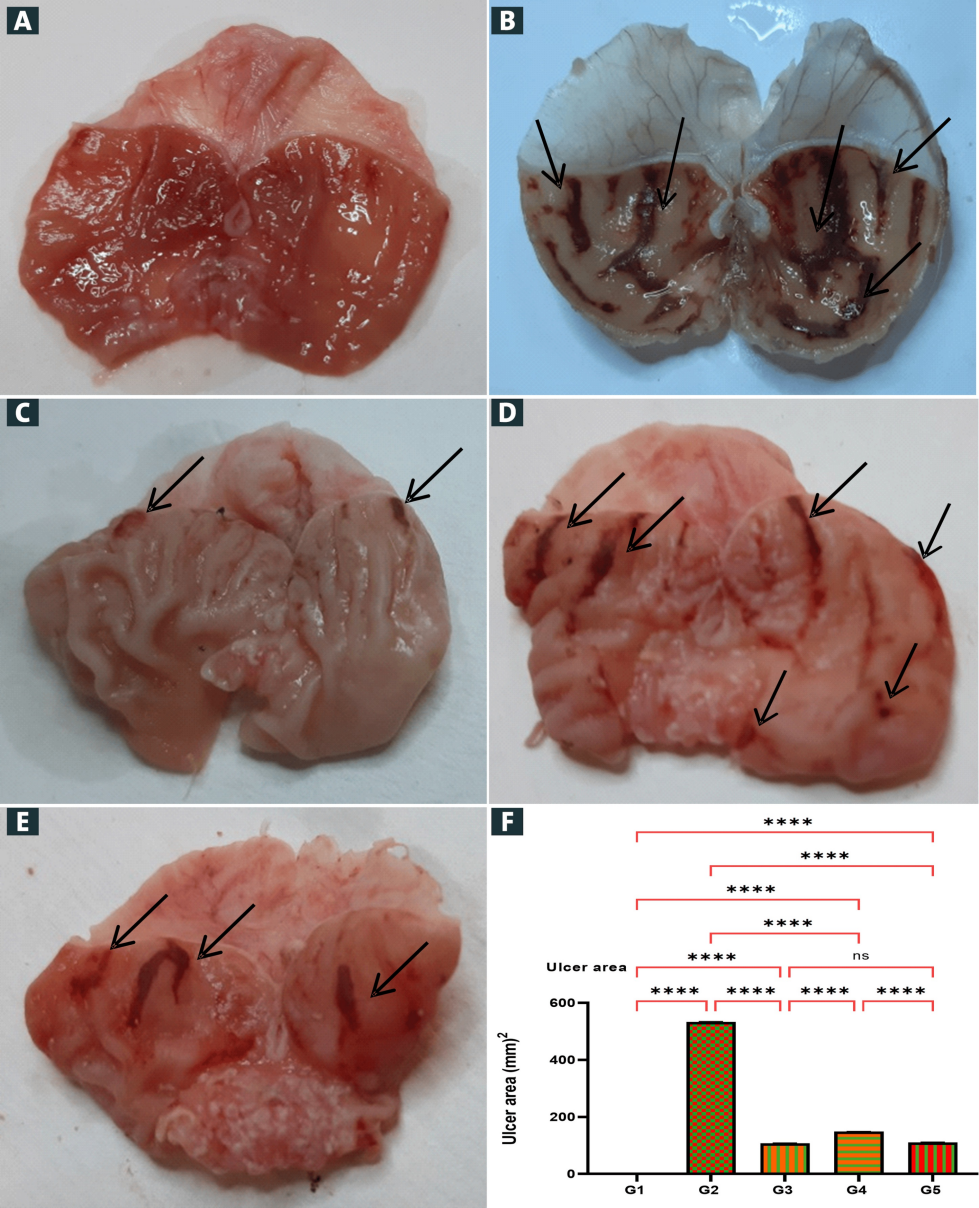
Macroscopic presence of gastric epithelium (A-E) and statistical analysis of ulcer area (F). (A) G1: Normal control showed undamaged stomach epithelium; (B) G2: Ulcer control had extensively damaged stomach mucosa (black arrows), absolute alcohol formed widespread noticeable hemorrhage to damage stomach mucosa; (C) G3: Omeprazole (20 mg/kg) damages stomach epithelium with very minor injuries (black arrows) compared to the ulcer control group; (D) G4: Schiff base compound (25 mg/kg) decreases the development of stomach lacerations induced by absolute alcohol (black arrows); (E) G5: Schiff base compound (50 mg/kg) minor damages stomach epithelial mucosa (black arrows). The statistical results indicated that Schiff base compound at dosages of 25 and 50 mg/kg, respectively, produced significantly different inhibition effects in preventing the formation of stomach ulcers to that of ulcer control (p<0.01) (G2) (F). ns, non-significant. ****p<0.0001.

Gastroprotective action of Schiff base compound against absolute alcohol-induced stomach abrasion model is presented in Figure 2. The results showed rats pre-fed with omeprazole or Schiff base compound meaningfully condensed parts of stomach ulcerated formation compared to the ulcer control group (Figures 2A-2F). Alcohol formed severe noticeable hemorrhagic lacerations and stomach epithelia. Furthermore, the Schiff base compound suggestively inhibited the development of ulceration. Rats pre-administered 50 mg/kg Schiff base compound protected stomach epithelium and, additionally, alcohol-induced epithelial injury. The alcohol ingestion created a higher gastric ulcer area in G2 (577.3 ± 7.47 mm^2^) than that 93.0± 2.60, 152.5 ± 6.71, and 104.0±6.66 mm^2^ of G3, G4, and G5 (p < 0.05), respectively.

Influence of Schiff base compound on stomach mucus content

Treat rats administered Schiff base compound presented a significant (p<0.05) increase in stomach mucus excretion compared to ulcer group (Table 5).

Influence of Schiff base compound pH gastric content

The pH stomach gratified investigational rats pre-treated with Schiff base compound or omeprazole was increased suggestively (p<0.05) compared to the ulcer control group (Table 5).

**Table 5:** Influence of Schiff base CdCl2 (C14H21N3O2) compound mucus mass, gastric pH, ulceration expanse, inhibition percent ulcer part alcohol-induced stomach ulcer. Mean value ± SEM (n = 6). ^a,b^Values indicated by different superscripts within the same column are significantly different according to Tukey’s honestly significant difference test at a p <0.05 significance level.

Animal Groups	Pre-feeding (5 ml/kg)	Mucus Weight (g)	pH	Ulcer Area mm^2^	Inhibition (%)
G1 normal control	10% Tween 20	2.57±0.13	7. 02±0.09	-	-
G2 ulcer control	10% Tween 20	0.59±0.03^a^	2.70±0.03^a^	577.3±7.47^a^	-
G3 Omeprazole	20 mg/kg Omeprazole	2.20±0.14^b^	6.450±0.27^b^	93.00±2.60^b^	87.71
G4 Schiff base compound	25 mg/kg Schiff base compound	1.61±0.021^b^	6.45±0.27^b^	152.5±6.71^b^	79.86
G5 Schiff base compound	50 mg/kg Schiff base compound	1.99±0.10^b^	6.41±0.19^b^	104.0±6.66^b^	86.26

Histological evaluation of stomach lesions

Hematoxylin and Eosin (H&E) Stain

**Figure 3:**
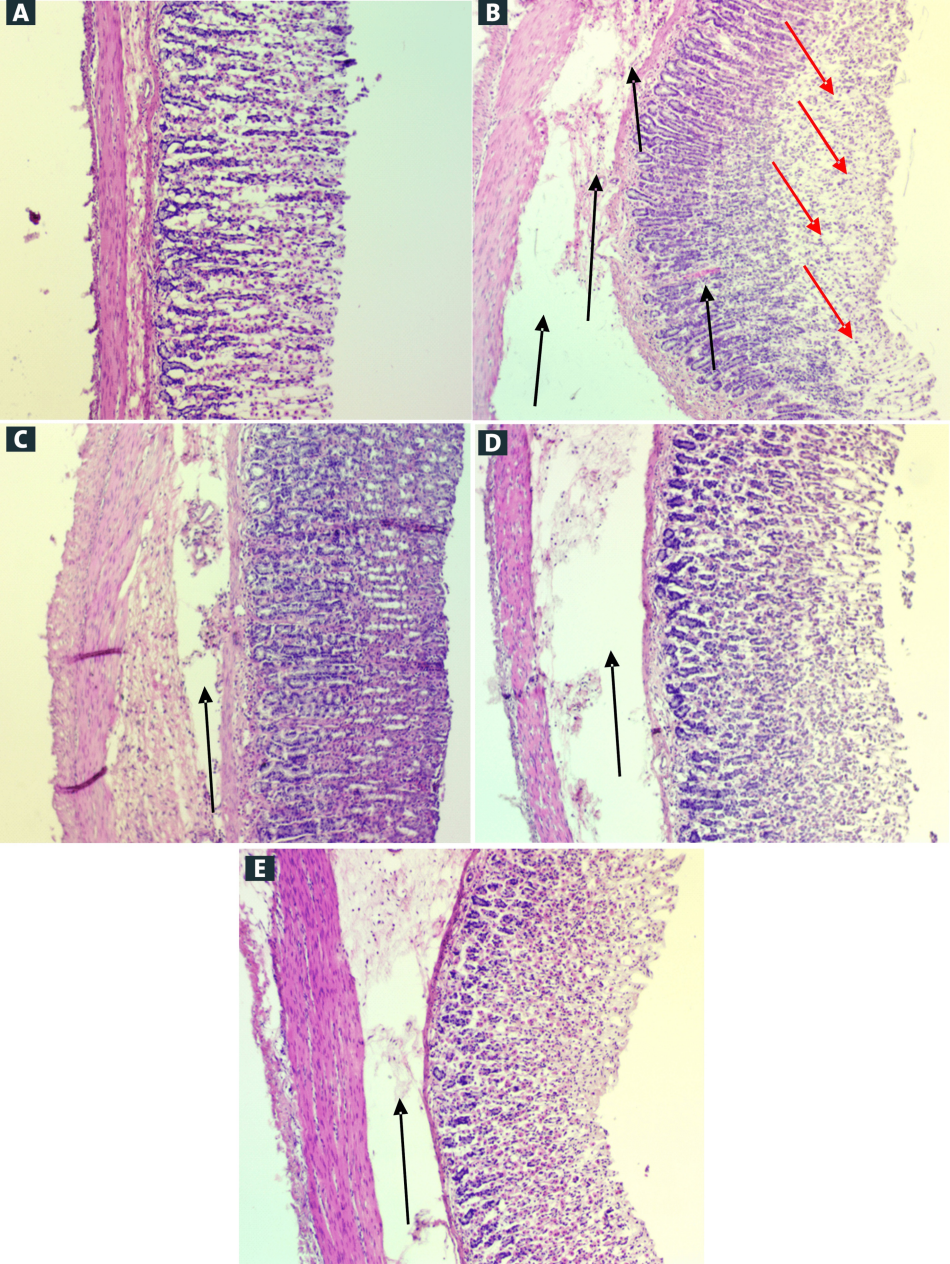
Effect of Schiff base compound on the histological study of the absolute alcohol-induced stomach mucosal injury. (A) G1: The normal group displayed intact stomach epithelia. (B) G2: Ulcer group pre-fed with 10% Tween 20. Extensive disturbance superficial mucosa dead lacerations infiltrate intensely mucosa (red arrows) widespread edema of submucosa coating inflammatory cells permeation existing (black arrows). (C) G3: Rats nourished omeprazole (20 mg/kg). Slight interruption of outward mucosa exists (black arrow); however, profound epithelial injury was absent. Decreasing of submucosal edema and white blood cell penetration (black arrow). (D) G4: Schiff base compound (25 mg/kg), minor disturbance surface epithelium existing then unfathomable mucosal impairment absent. Decrease submucosal edema inflammatory permeation (black arrow). (E) G5: Schiff base compound (50 mg/kg), minor disturbance surface epithelium existing nonetheless profound mucosal injuries is inattentive. Decline submucosal edema and inflammatory penetration (black arrow) (hematoxylin and eosin (H&E) stain, magnification 20×).

Histology remark absolute alcohol stimulated stomach injuries ulcerated control group pre-fed with 10% Tween 20, presented moderately wide-ranging destruction of stomach mucosa necrotic lacerations penetrate intensely epithelium, and wide edema and inflammatory cells penetration sub-epithelial layer existing (Figure 3). Rats pre-treated with the Schiff base compound required moderately improved defense stomach mucosa via decreasing ulcer region and condensed sub-epithelial edema and white blood cells permeation (Figures 3A-3E). Schiff base compound revealed the use of cytoprotective of stomach mucosa against ethanol-induced gastric ulcer in rats.

Periodic Acid-Schiff (PAS) Stain

**Figure 4:**
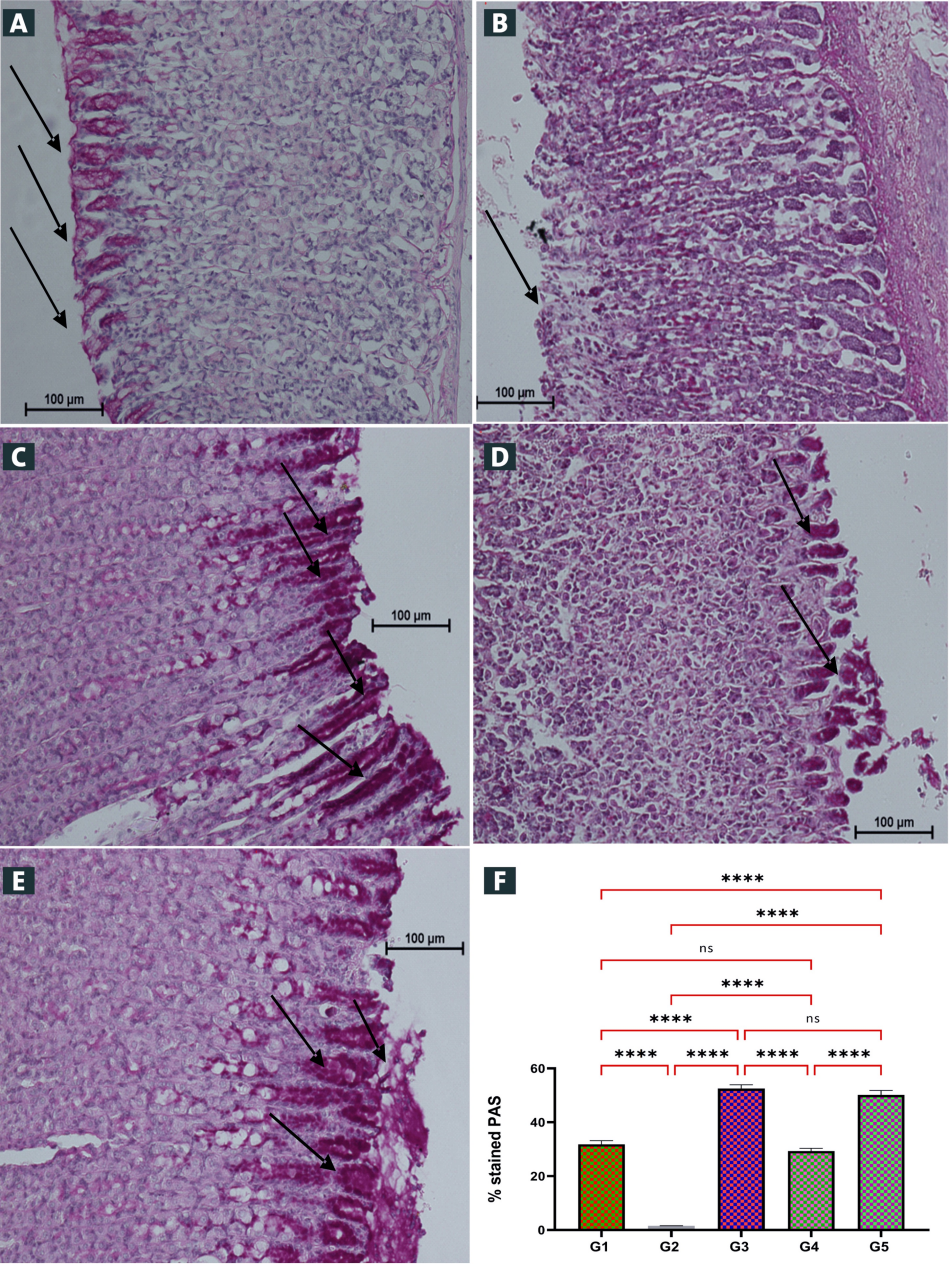
Influence of the Schiff base compound on stomach material glycoprotein-PAS stained (A-E) and statistical analysis (F). (A) G1: The control presented undamaged stomach epithelium. (B) G2: Ulcer control exhibited sever stomach tissue damage with very appearance of magenta PAS stain, (C) G3: Omeprazole group (20 mg/kg) had non-significant stomach tissue disruptions with a high PAS staining concentration. (D) G4: Schiff base compound 25 mg/kg mild to moderate damage of gastric mucosa with mild PAS stain of gastric mucosa. (E) G5: Schiff base compound 50 mg/kg mild damage of gastric mucosa and with moderate PAS stain of gastric mucosa. Magenta color apical epithelial cells of Schiff base compound administered group’s demonstrations regularly increase mucosal excretion stomach glands (black arrow). The powerful excretion of mucus stomach glands confirmed (E). The statistical analysis indicated that the Schiff base compound of dose (50 mg/kg), and omeprazole group showed the highest PAS stained percentage as significant different to that of the ulcer control group (G2) (p<0.01) (F). The arrow points to the glycoprotein gathering (PAS stain, magnification 20×). PAS: periodic acid-Schiff; ns: non-significant. ****p< 0.0001.

PAS stains are utilized to detect glycogen quantity of the stomach mucosa. Schiff base compound pre-treatment (Figure 4) caused the development of a significantly incessant PAS-positive mucous gel sheet coating the whole stomach epithelial superficial observed magenta color. However, the stomach mucosa of ulcer control group did not display a magenta stain color PAS (Figures 4A-4F). Statistical analysis revealed the percentage of PAS stains in all animal groups (Figure 4F). Rats treated with Schiff base compound had higher PAS staining concentrations of the gastric glycoprotein than those of the negative (ulcer) control (Figure 4F). The ulcer control group showed significantly greater gastric tissue disruption with severe inflammatory edema and leukocytes than those of the treated rats.

Immunohistochemically stain

Expression of HSP-70 Protein

**Figure 5:**
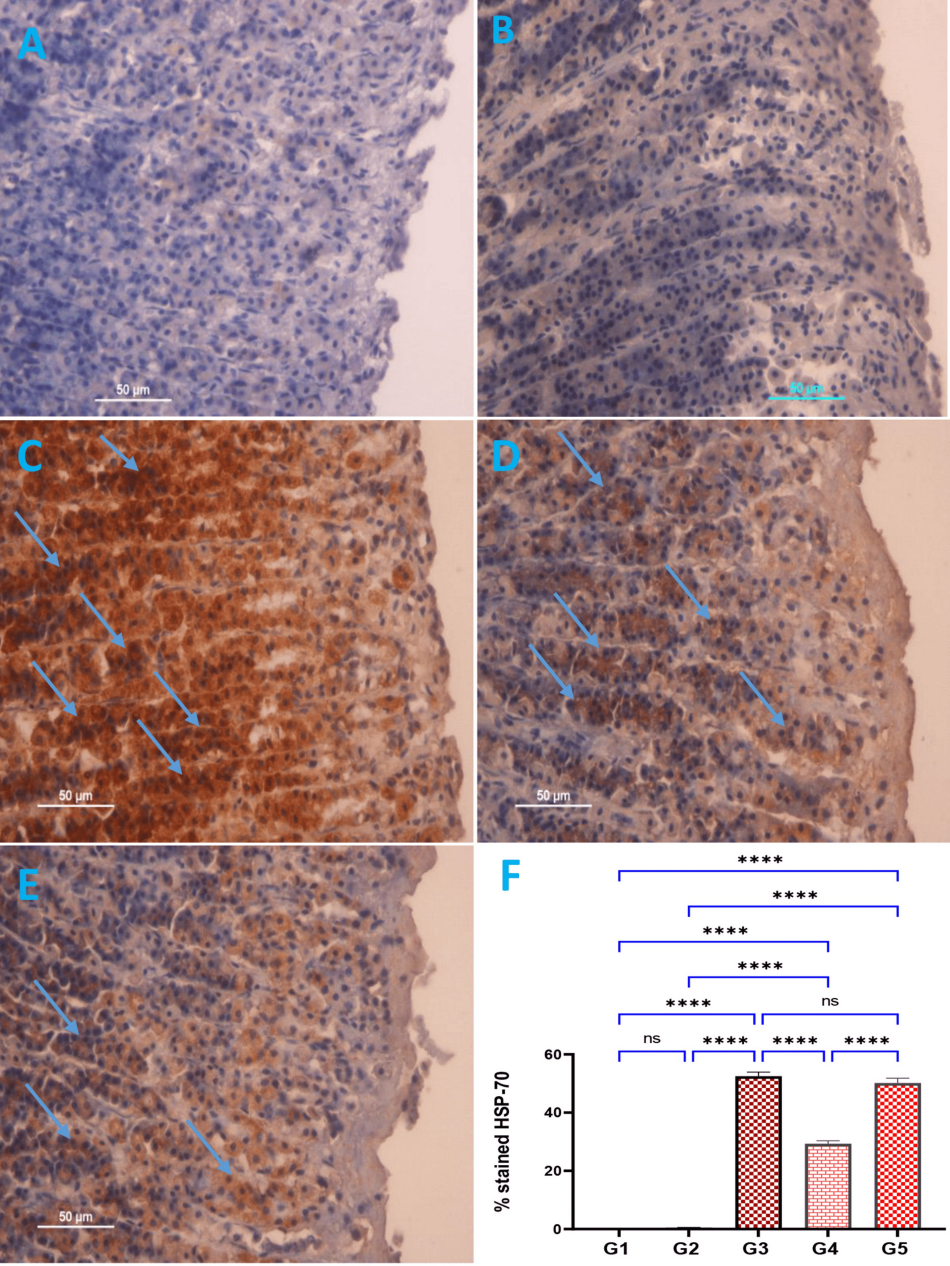
Influence of Schiff base compound on HSP-70 protein expression of alcohol-induced stomach epithelial grazes in diverse groups of animals (A-E) and quantitative analysis (F). G1: Normal control exhibited complete stomach mucosa and very weak HSP-70 protein expression in the gastric mucosa layer (A, F), G2: Ulcer control group presented severe injury of the gastric mucosa and down-regulation of HSP-70 protein expression in alcohol-induced gastric ulcers in rats compared to the other treated groups (B, F). G3: Omeprazole group exhibited mild damage of the gastric mucosa and up-regulation of HSP-70 protein expression of gastric mucosa in alcohol-induced gastric ulcers in rats (blue arrow) (C, F), G4: Rats administered Schiff base compound 25 mg/kg had moderate stomach lesions (blue arrow) with increased representations of HSP-70 in their stomach tissues (D, F). G5: Rats administered Schiff base compound 50 mg/kg showed a mild stomach tissue injury (blue arrow) with increased representations of HSP-70 protein, compared to the ulcer-controlled rats (E, F). The antigen place seemed brown (HSP-70 stain magnification 20x). Values expressed as means ± SEM. The antigen area is observed in a brown color. The significant difference in the HSP-70% protein expressions are presented as ns. ns: non-significant;  HSP-70: heat shock protein-70. *p < 0.05; ****p < 0.0001.

Using immunohistochemistry staining, the immuno-stained confine HSP-70 significantly up-regulated in Schiff base compound pre-treated animals and down-regulated ulcer control group (Figure 5). The appearance of HSP-70 protein stain showed strong brown color stains antigen in rats pre-treated omeprazole and Schiff base compound (Figures 5A-5F). The analysis of the percentage of HSP-70 stained protein expressions displayed antigens with a strong brown color in rats administered with omeprazole (G3) or Schiff base compound (G4 and G5) (Figure 5F).

Expression of Bax Protein

**Figure 6:**
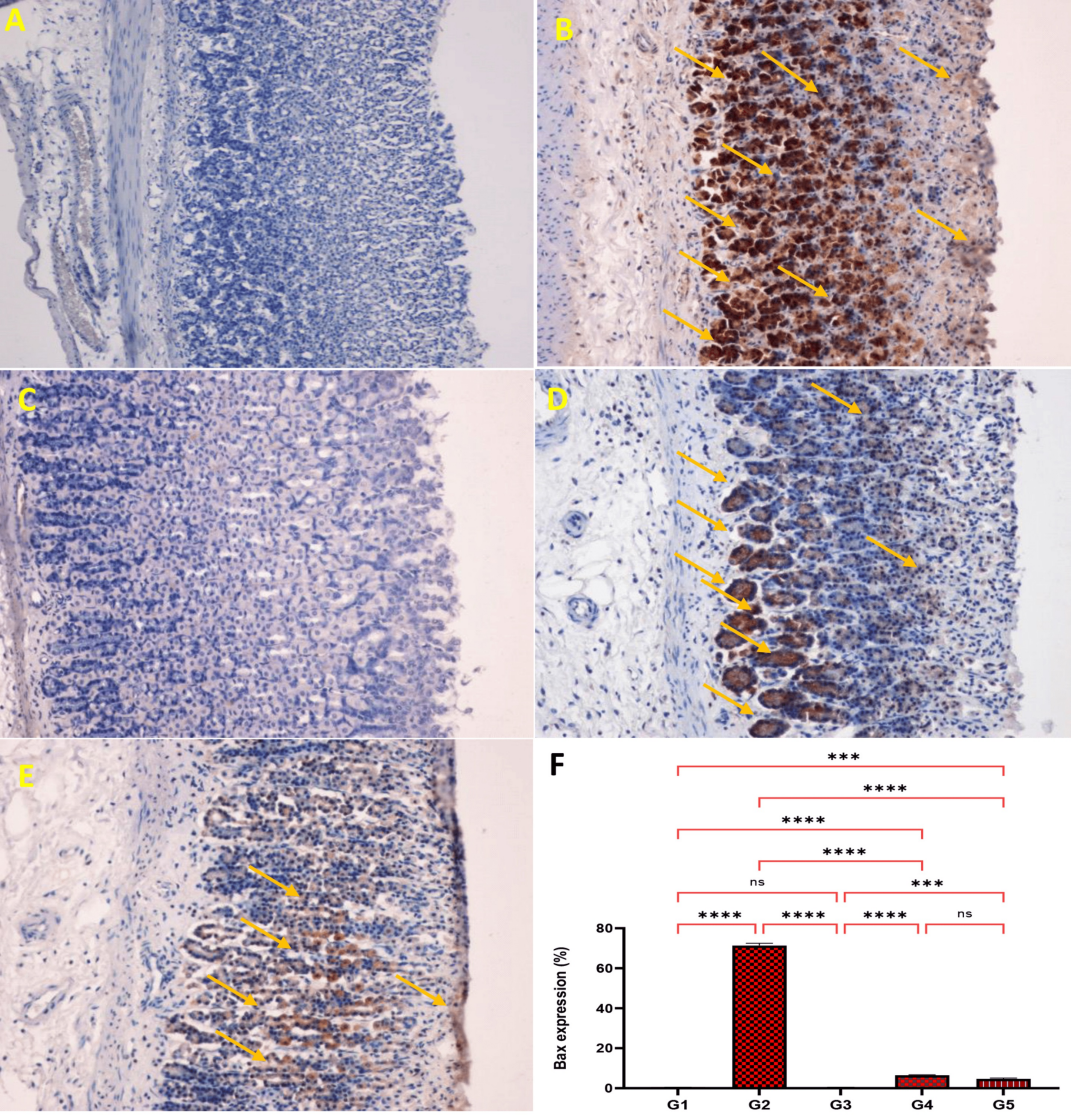
Influence of Schiff base compound on Bax protein expression alcohol-induced stomach epithelial lesions in different groups of rats (A-E) and statistical analysis (F). G1: The normal control displayed unharmed stomach mucosa and very weak expression of Bax protein in the gastric mucosa (A, F). G2: The ulcer control group presented severe damage to the gastric mucosa and up-regulation of Bax protein expression in alcohol-induced gastric ulcers in rats (B, F). G3: The omeprazole group showed mild damage of the gastric mucosa and down-regulation of Bax protein expression of the gastric mucosa  (orange arrow) compared to the ulcer control group in alcohol-induced gastric ulcers in rats (C, F). G4: Schiff base compound 25 mg/kg showed moderate injury of the gastric mucosa and down-regulation of Bax protein expression of the gastric mucosa (orange arrow) compared to the ulcer control group in alcohol-induced gastric ulcers in rats (D, F). G5: Schiff base compound 50 mg/kg displayed mild damage of the gastric mucosa and down-regulation of Bax protein expression of the gastric mucosa (orange arrow) compared to the ulcer control group in alcohol-induced gastric ulcers in rats (E, F). The antigen position looks brown (Bax stain magnification 20x). There was significant differentiation between rat groups in Bax protein expressions with the highest expressions recorded for the G2 rat group. ns: non-significant; Bax: Bcl-2-associated X protein. ***p<0.001. ****p<0.0001.

Bax protein is significantly down-regulated in Schiff-base compound pre-treated groups and up-regulated in ulcer control group. Antigen location Bax protein expression seems brown colored in the ulcer control group (Figures 6A-6F). Statistical analysis revealed that G2 ulcer group showed highly significant Bax protein expression percentage as compared with omeprazole and two doses of the Schiff base compound groups (Figure 6F).

Effects Schiff base compound on antioxidant enzymes on SOD and CAT

**Figure 7:**
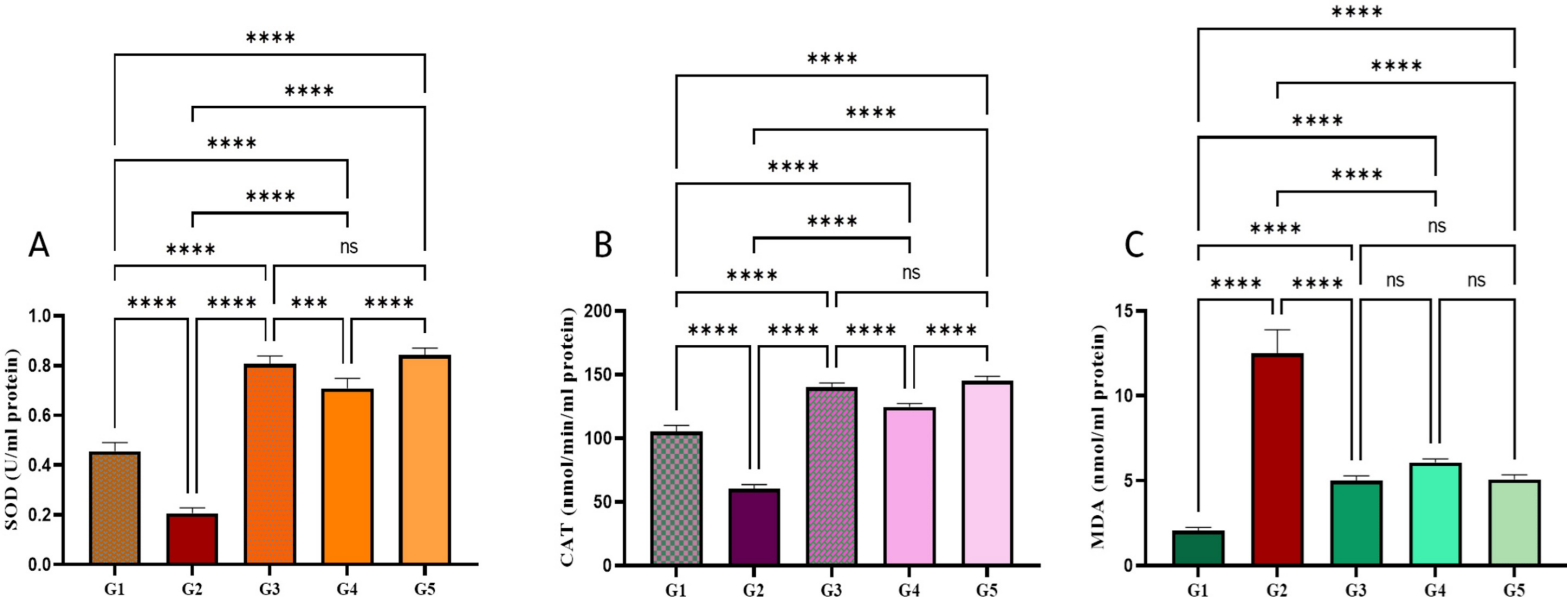
The Effect of Schiff base compound on SOD, CAT, and MDA in absolute alcohol prompted stomach ulcers. Pre-treatment groups: G1: normal control (10% Tween 20). G2 ulcer control (10% Tween 20), G3 Omeprazole 20 mg/kg, G4 Schiff base compound 25 mg/kg. G5 Schiff base compound 50 mg/kg. Data are expressed as mean ± SEM. Means among groups (n = 6 rate/group) show a significant difference. ns, non-significant, SOD: superoxide dismutase, CAT: catalase, MDA: malondialdehyde. *** p < 0.001. **** p < 0.0001.

The ulcer control group showed significantly reduced SOD and CAT activities compared to normal group (Figure 7). Rats nourished with Schiff base compound obtainable knowingly increased SOD and CAT activities, achieving usual values. Rats fed with Schiff base compound 50 mg/kg had suggestively greater SOD and CAT standards compared to the rats fed with Schiff base compound 25 mg/kg and ulcer control groups.

Effects of the Schiff base compound on malondialdehyde (MDA)

Ulcer control gathering presented a considerable upsurge in MDA levels compared to normal group (Figure 7). MDA level knowingly reduced rats nourished Schiff base compound. MDA indicator of lipid peroxidation (Figure 7).

Effect of the Schiff base compound on cytokines level in blood

**Figure 8:**
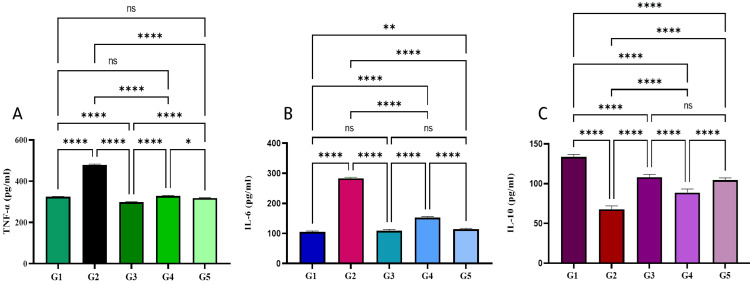
Influence of Schiff base compound on TNF-α, IL-6, and IL-10 in alcohol-induced stomach ulcers. G1: normal control (10% Tween 20). G2: ulcer control (10% Tween 20), G3 Omeprazole 20 mg/kg, G4 Schiff base compound 25 mg/kg. G5 Schiff base compound 50 mg/kg. Data are expressed as mean ± SEM. Means among groups (n = 6 rate/group) show a significant difference. ns, non-significant. *p < 0.05. **p < 0.01. ****p < 0.0001. TNF-α: tumor necrosis factor-alpha; IL-6: interleukin-6.

Results in inflammatory cytokine investigation are displayed in Figure 8. TNF-α, IL-6, and IL-10 are significantly higher in ulcer control group compared to the fed with omeprazole or Schiff base compound. Rats treated with omeprazole or Schiff base compound meaningfully decreased TNF-α and IL-6. Augmented IL-10 was compared to the ulcerated control (Figure 8).

## Discussion

The acute toxicity properties of the Schiff base compound did not exhibit harm or death throughout the experiment, when orally gavage (250 and 500 mg/kg). There was no evidence of poisonousness in the liver and kidney during the biochemical and histological parameters. Numerous studies on chemical compounds reported no hepatic and renal toxicity in rats [2,3,18].

Absolute alcohol gavage produced wide-ranging disturbance gastric mucus fence, accompanied by weakened mucous discharge. Moreover, absolute alcohol increased microvascular penetrability and provoked lipid peroxidation. Furthermore, alcohol produced free-radical construction capabilities of gastric mucosa and then produced widespread gastric mucosal injury [1,4,19]. The results established that the Schiff base compound protects the mucosa by secreting extra mucous. Similarly, various studies using the Schiff base complex reported gastroprotective due to enhanced mucus secretion [3,19-21].

In the present study, experimental rats fed with the Schiff base compound exhibited flattened stomach mucosal crinkles, improved mucosal superficial part, and lessened gastric damage than the ulcerated control group. Likewise, numerous researchers utilizing chemical compounds described flattened stomach epithelial and enhanced gastro-defend influences in laboratory animals [1,15,19].

Histological investigation of the gastric ulcer control group showed severe hemorrhagic stomach damage improved white blood cell infiltration triggering edema submucosal layer. Correspondingly, investigational rat groups nourished Schiff base complexes revealed gastro protective [2]. The results of contemporary trials discovered rats nourished Schiff base complexes displayed improved intensity PAS stains compared to the ulcer control group. Likewise, numerous studies utilized diverse Schiff base compound; remedial plants reported enhanced forte PAS stains in stomach sections in experimental investigational rats [1,2,22].

The present study showed ethanol-increased construction ROS causing stomach epithelial impairment in rats. ROS resulted in the down-regulation of HSP-70 and increased pro-apoptotic Bax protein expression [1,2,23]. HSP-70 proteins protect stomach mucosa from oxidative stress-induced absolute ethanol preventing partly denatured proteins assembly. Up-regulated HSP-70 investigational groups nourished the Schiff base compound-directed defense gastric wall. Several researchers have designated that the up-regulated HSP-70 trial rats nourished synthesized chemical compounds against ethanol-prompted stomach ulcers, through declining ROS-mediated stomach oxidative pressure [1-3,23]. HSP-70 has the competence to avoid manufacturing oxidative stress-induced absolute ethanol. Significant gastroprotective effects of HSP-70 were reported in previous studies [1,23]. HSP-70 characteristically underway cytoplasm, nucleus, mitochondria, cell membranes, and extracellular space. Its existence increases at shares cellular tension and defends compared to injury. In stomach ulcers, HSP-70 defense comprises expectant normal protein construction during the elimination of impaired protein [5,24].

Bax proteins are a member of Bcl-2 family [2,25]. Absolute alcohol causes the beginning apoptosis of gastric mucosa through over appearance of pro-apoptotic protein. Bax encourages down countenance anti-apoptotic forms, for example, Bcl-2 [1,2]. Rats fed with Schiff base compound revealed down-regulated Bax protein and up-regulated HSP-70 proteins in gastric tissue sections compared to ulcer control collection. Outcomes with the contract consequence preceding research are stated in various studies [1,2].

The anti-ulcer activity of Schiff base compound reduced ethanol-induced stomach injury and declining inflammatory cell infiltration submucosal layer. With the consistence of the outcomes in existing research, several investigators described anti-inflammatory besides anti-ulcer action Schiff base compound [21, 26]. The antioxidant efficacy of Schiff base compound by up-regulated SOD and CAT and then down-regulated MDA levels was stated in two studies [26,27].

The findings of the present investigation displayed rat-fed Schiff base compound significantly reduced MDA and increased SOD and CAT in stomach epithelial homogenates prevent oxidative pressure induced by the absolute alcohol-forced feeding. Correspondingly, the vast number of searchers has described several detectives applying curative plants, Schiff base complexes, or synthetic compounds, which disclose increased enzyme undertakings (SOD and CAT) and reduced MDA quantity stomach homogenates [19]. SOD and CAT provide significant stomach epithelial protection, prevent oxidative stress, and inspire gastric repair, thus decreasing ROS and encouraging gastric protection. Abundant quantities of SOD and CAT in the stomach wall eliminate free radicals made by absolute alcohol [3]. The result of the current study revealed Schiff base complexes well-maintained gastric SOD and CAT-reducing MDA [19]. Schiff base compound significantly protects gastric mucosa from the toxic effects of ethanol through the improved endogenous enzymes (SOD and CAT) and condensed MDA stomach homogenate. With the consistent result of the results of the current study, several investigators using synthetic compounds or medicinal plant extracts reported the rise of endogenous enzymes and reduction of lipid peroxidation (MDA) in ethanol-induced stomach ulceration [1,2,4].

TNF-α, IL-6, and IL-10 are secreted via macrophages, a pronounced significant role in ethanol-induced gastric injury via neutrophil infiltration submucosal layer [1,2]. In the current study, Schiff base compound condensed amount TNF-α and IL-6, enhancing IL-10 peripheral blood. Rats treated with Schiff base compound meaningfully condensed TNF-α and IL-6, increased IL-10 quantities, and related ulcer control group. IL-6 stimulates neutrophils and mononuclear cell location irritation [2]. IL-6 encourages the formation of the greatest acute phase proteins.

During free radical scavenging, contrivance performance of the Schiff base compound could be decreasing the development of only oxygen, protecting stomach epithelia contrary to oxidative pressure, and stimulating gastric restoration and anti-inflammatory apparatuses.

Limitations of the research

The current research has some limitations; in the histopathological evaluation, another staining method could be used to see the variability in the compound microscope, Alcian blue stain (for glycoprotein in the mucus), and Feulgen stain (for nucleus) that can be used instead of H&E stain.

## Conclusions

Schiff base compound does not cause acute harmful effects in rats (250 mg or 500 mg/kg). The findings of the current investigations showed Schiff base compound exposed expressively gastro protective influence ethanol-induced gastric damage investigational rats, recognized by macroscopically, and histological examination. Schiff base compound improved gastric mucus discharge, flattened stomach mucosa, increased pH gastric content, and reduced edema inflammatory cell infiltration of the submucosal stratum stomach wall. Stomach mucosal homogenates and Schiff base compound significantly up-surged SOD and CAT activities and significantly reduced the MDA level. Besides, the Schiff base compound induces up-regulated HSP-70 proteins plus down-regulated Bax proteins in stomach epithelia slices in investigational rats. Defensive significances are mostly attributed to antioxidant as well as anti-inflammatory intermediaries in gastric walls. Schiff base compound might during free radical hunting plummeting building sole oxygen, thus defensive stomach epithelium against oxidative stress ethanol, hopeful stomach epithelial renovation anti- inflammatory machinery. Schiff base compound recovers acute stomach ulcers encouraged via ethanol in rats, which could be mostly due to its antioxidant capability besides anti-inflammatory influences.

## Figures and Tables

**Figure 9:**
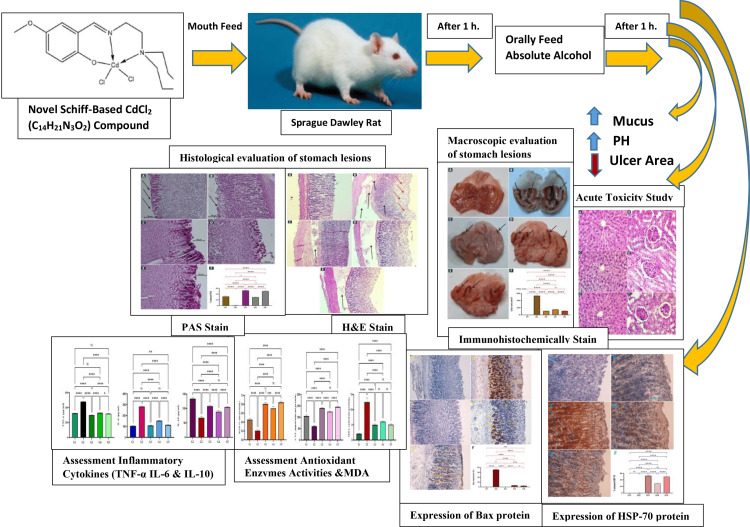
Graphical abstract: in vivo gastroprotective upshots of novel Schiff base CdCl2 (C14H21N3O2) compound. PAS: periodic acid-Schiff; TNF-α: tumor necrosis factor-alpha; IL-6: interleukin-6; HSP-70: heat shock protein-70; Bax: Bcl-2-associated X protein; MDA: malondialdehyde.
